# Emotions in Covid-19 Twitter discourse following the introduction of social contact restrictions in Central Europe

**DOI:** 10.1007/s10389-021-01613-y

**Published:** 2021-07-02

**Authors:** Franz Hanschmidt, Anette Kersting

**Affiliations:** Department of Psychosomatic Medicine, University of Leipzig, Semmelweisstraße 10, 04103 Leipzig, Germany

**Keywords:** Natural language processing, Social media, Covid-19, Anxiety, Emotions, Topic models

## Abstract

**Aim:**

Non-pharmaceutical interventions such as lockdowns have played a critical role in preventing the spread of the Covid-19 pandemic, but may increase psychological burden. This study sought to examine emotions reflected in social media discourse following the introduction of social contact restrictions in Central Europe.

**Subjects and methods:**

German-language Twitter posts containing ‘#corona’ and ‘#covid-19’ were collected between 2020/03/18 – 2020/04/24. A total of 79,760 tweets were included in the final analysis. Rates of expressions of positive emotion, anxiety, sadness and anger were compared over time. Bi-term topic models were applied to extract topics of discussion and examine association with emotions.

**Results:**

Rates of anxiety, sadness and positive emotion decreased in the period following the introduction of social contact restrictions. A total of 16 topics were associated with emotions, which related to four general themes: social contact restrictions, life during lockdown, infection-related issues, and impact of the pandemic on public and private life. Several unique patterns of association between topics and emotions emerged.

**Conclusion:**

Results suggest decreasing polarity of emotions among the public following the introduction of social contact restrictions. Monitoring of social media activity may prove beneficial for an adaptive understanding of changing public concerns during the Covid-19 pandemic.

## Introduction

After a period of rapid global spread, the World Health Organization (WHO) declared the coronavirus disease 2019 (Covid-2019) outbreak a global pandemic on 11 March 2020 (World Health Organization 2020). The first cases were observed in China in December 2019, and Covid-19 had infected 9,457,902 people worldwide by May 2020, causing an estimated 482,247 deaths (John Hopkins University 2020). The pandemic has put serious strain on healthcare systems given the high infection and transmission rates, the lack of efficient treatment options and the clinical severity of Covid-19 (Lipsitch et al. 2020). To prevent transmissions, several countries implemented a range of non-pharmaceutical interventions aiming at restricting social contacts, such as social distancing rules or lockdowns (Hsiang et al. 2020; Cheng et al. 2020). Countries in Central Europe (e.g. Germany, Austria, Switzerland) introduced comprehensive restrictions over a short period from the mid to end of March 2020, which largely prohibited social contacts, forced people to stay at home and led to nation-wide shutdowns of educational institutions and the public business sector (Hale et al. 2020). The psychological and emotional correlates of such comprehensive restrictions of social contact remain largely unexplored.

Adverse emotions experienced in response to a pandemic have been associated with increased psychological burden, unhealthy behaviours, and biased risk perception (Asmundson and Taylor 2020; Lin et al. 2020; Saltzman et al. 2020). Thus, understanding the emotional implication of the Covid-19 pandemic for the public is important for tailoring public health messages and service provision to people’s needs. Anxiety has been identified as a central emotional response to the high risk of infection and the uncertainty related to the unpreceded circumstances during the Covid-19 pandemic. Several studies suggest that rates of anxiety, depression and stress are elevated in populations affected by Covid-19 (Luo et al. 2020). In a German survey, 60% and 74% of respondents reported being anxious and worried because of Covid-19 at the time the restrictions of social contact were announced (Betsch et al. 2020). Efforts aimed at limiting the spread of Covid-19, such as lockdowns and quarantine, often severely restrict people’s capacities to access social and economic resources, which may result in anger and frustration (Brooks et al. 2020; de Las Heras-Pedrosa et al. 2020). Other study results indicate that positive emotions may emerge from increased feelings of support, e.g. from friends and family (Zhang and Ma 2020).

Information available on social media platforms such as Twitter constitute an important resource for monitoring emotional responses at different stages of the pandemic. A number of studies have analysed large bodies of Twitter posts (‘tweets’) to advance knowledge regarding public opinion, experiences of stigma or emotions related to Covid-19 (Budhwani and Sun 2020; Jimenez-Sotomayor et al. 2020; Rufai and Bunce 2020; Han et al. 2020). Findings of a study of tweets from English-language users suggests that common topics discussed on Twitter in the earlier phase of the pandemic include the origin of the virus, its socio-economic impact and risk of infection (Abd-Alrazaq et al. 2020). Most of the topics were characterized by positive emotions. In another analysis of English-language tweets, Lwin et al. found that emotions reflected in narratives on Twitter were dominated by fear related to disease spread in the beginning of the pandemic, and shifted to anger associated with social isolation as the pandemic progressed (Lwin et al. 2020). Language indicative of sadness was used in tweets related to loss of loved ones due to a Covid-19 infection, while positive emotion were expressed in tweets associated with gratitude, hope and resilience (Lwin et al. 2020).

These studies highlight the potential of using social media data to discover changing emotions and concerns of the public over the course of the Covid-19 pandemic. However, many available studies have analysed tweets posted by twitter users globally, representing a heterogeneous population with regard to exposure to infection risk and public health efforts to control the spread of the disease. Examining region-level social media discourse may allow for a more nuanced assessment of psychological and emotional responses of the public at different stages of the pandemic. A study that compared social media posts originating from regions of China and Italy before and after the lockdown found different patterns of language-use reflecting different emotional responses (Su et al. 2020). Anxiety appeared to decrease in Italy following the introduction of social contact restrictions, whereas negative emotions remained unchanged in China (Su et al. 2020).

Several countries in Central Europe such as Germany, Austria and Switzerland simultaneously introduced comparable social contact restrictions over a short period from the mid to end of March 2020 (Hale et al. 2020). Public responses to these measures can be readily identified in social networks such as Twitter by focusing on German language use, which is used only in a confined geographical area in Central Europe covering mainly the populations of Germany, Austria and Switzerland (Eberhard et al. 2019). Thus, the aim of this study was to examine trends and topics associated with emotions reflected in German Covid-19-related discourse on Twitter following the introduction of social contact restrictions in countries of German-speaking Central Europe. Results can be instructive for a more effective coordination of policy and health system response to the psychological outcomes of the Covid-19 pandemic.

## Methods

### Data collection and cleaning

Twitter’s standard search API, which searches against a sample of tweets published in the past 7 days, was used for data collection. In a series of consecutive searches, German-language tweets that (a) contained the terms ‘#corona’, ‘#coronavirus’, ‘#covid19’ or ‘#covid’; and (b) were published between 2020/03/18 and 2020/04/24 were collected. User-tags and geo-location of tweets were inspected to determine region of tweet origin.

Only tweets containing a self-reference (i.e. ‘I’, ‘my’, ‘mine’, ‘me’) were selected for further analysis to increase the probability of capturing expressions of individual emotions. Tweets were prepared for analyses in several steps: (1) Duplicates were removed in a two-stage process. First, duplicates were identified by inspecting the ‘is_retweet’ label provided by Twitter for each tweet. Second, as some retweets may not have been correctly labelled for technical reasons, the cosine similarity of tweets was additionally calculated to identify duplicate tweets; (2) The position-of-speech of words within tweets was determined; (3) Basic cleaning of tweets was conducted by removing URLs, usernames and converting all characters to lower case.

Data collected in this study were made publicly available by Twitter users, and were thus treated as data within the public domain (Stevens et al. 2015). User anonymity was ensured by presenting data in aggregated form only. Therefore, no further ethical approval was deemed necessary for this study. Data usage and processing in this study adhered to Twitter’s Terms of Service and Developer’s Agreement and Policy.

### Materials

The German version of Linguistic Inquiry and Word Count (LIWC) dictionary was used to categorize words within tweets into four emotional categories based on previous research: positive emotion, anger, anxiety and sadness (Meier et al. 2019; Pennebaker et al. 2015; Lwin et al. 2020; Brooks et al. 2020; Luo et al. 2020). The LIWC dictionary has been specifically developed to capture an individual’s psychological states as reflected in language use. It contains selected words which are organized into a set of psychological categories (e.g. positive emotions, anxiety). For a given document, the rate of word counts in the respective LIWC category to the total number of words (%) is calculated, with higher rates reflecting more pronounced emotion. Reliability of the German version of the LIWC has been demonstrated and meaningful associations with related psychological constructs in a number of studies indicate validity (Meier et al. 2019).

### Data analysis

Sample characteristics were summarized using descriptive statistics (median, mean [M] and standard deviation [SD]).

Hashtags were removed before calculating rates of emotions as they are not a regular part of general language syntax and may bias word counts. To estimate changes in prevalence of emotions over times, tweets published on the same day were aggregated into a single document and rates of emotions were calculated. Time trends were modelled using logistic regression with day as independent and daily rate of emotion as the dependent variable.

In order to determine associations between emotions and tweet content, bi-term topic models were used to discover clusters of tweets with related content (Yan et al. 2013). A bi-term model (BTM) is based on the underlying assumption that word co-occurrences (i.e. bi-terms) within a set of documents (e.g. tweets) are generated by a mixture of probability distributions of unobserved topics (Yan et al. 2013). BTMs are especially suited to extract topics from short texts with sparse word co-occurrences like tweets (Yan et al. 2013). During the modelling process, words are probabilistically assigned to topics and these topic-word distributions allow for qualitative interpretation of the content of topics.

Topic models based on nouns-only have been shown to produce results which are more interpretable (Martin and Johnson 2015). Thus, only nouns, proper nouns and hashtags that (a) occurred in at least ten tweets; and (b) consisted of at least three characters were included in the topic modelling process. Additionally, we inspected the 100 most frequent words of this reduced corpus. We removed or merged terms that were judged to carry poor discriminative meaning and would thus occur in many topics (see S1 for sample flowchart). To guide decisions on the number of topics to extract, we calculated semantic coherence and exclusivity as metrics of topic quality as proposed in Roberts et al. (2019). Semantic coherence measures how well words assigned to a topic reflect the empirical co-occurrence of words within a set of documents (e.g. tweets) (Mimno et al. 2011). Exclusivity is based on a variant of the FREX metric (Bischof and Airoldi 2012), which assigns higher scores to topics containing words that are both frequent and exclusive to a given topic. We used formulas and code as provided by Roberts et al. (2019).

We ran a series of models with k-topic (k = 10,20,30,40,50,60,70,80,90,100) and selected the final model based on assessment of semantic coherence and exclusivity. Parameters estimated by the final model were used to assign each tweet its most likely topic. Associations between each topic and rates of emotions were calculated using logistic regressions with topic as dependent variable and rates of emotion as independent variables. Only topics showing a positive association with any emotion were retained. Finally, topics were labelled and interpreted by inspecting the topic-word distributions returned by the model. Two metrics were used to identify top words associated with each topic: probability and FREX. Probability is the statistical probability for a word to belong to a given topic, whereas the FREX metric weights the probability of words by their exclusivity for a given topic. Words which are both probable and exclusive score higher on the FREX metric (Roberts et al. 2019). Additionally, we inspected most likely tweets associated with each topic.

All data analysis was conducted using R (R Core Team 2019). Results with a *p* value < .05 or a confidence interval that did not contain the null hypothesis were considered significant.

## Results

### Sample characteristics

Sample characteristics are displayed in Table 1. A total of 608,737 tweets were collected (no data collected on 2020-04-17). After removing duplicates and selecting tweets with self-reference, 79,760 tweets were included in the analysis (see S1 for sample flowchart). The mean number of tweets posted per day was M = 2156 (SD = 914). Tweets were posted by 37,020 unique users with the mean number of tweets posted by a single user being M = 2.15 (SD = 3.88). Information on user-tagged country of origin of tweets was available for 3.8% (*n* = 3034) of the sample. Of these, 97.2% were tagged with Germany (85.4%, *n* = 2590), Switzerland (6.1%, *n* = 185) or Austria (5.7%, *n* = 173) as the country of origin. Geo-location was enabled for 0.2% of all tweets only.

**Table 1:** Sample characteristics

Characteristic	Statistic
Sampling period	2020/03/18 – 2020/04/24
Number of tweets	79270
Mean (SD) number of tweets per day	2156 (SD = 914)
Number of unique users	37,020
Mean (SD) number of tweets by single users	2.15 (SD = 3.88)
User country of origin (n[%])	
Germany	2590 (3.25)
Switzerland	185 (0.23)
Austria	173 (0.22)
Other	86 (0.11)
Missing	76726 (96.2)

### Rates of emotions

**Fig. 1:**
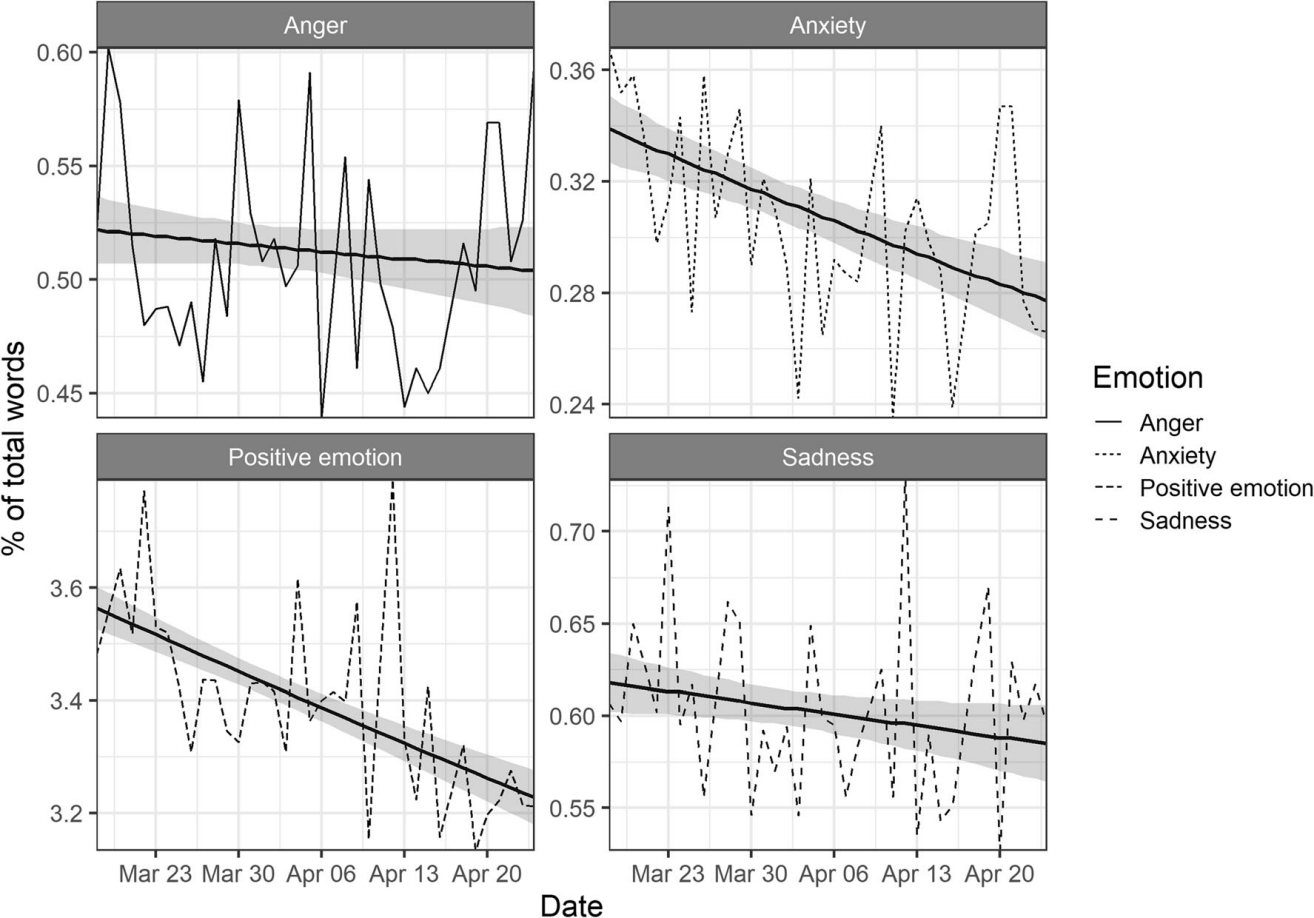
Rates of emotions in Covid-19 related tweets by day. Trends lines and 95% confidence interval fitted with logistic regressions

Figure 1 shows the rates of different emotions by day and the fitted trend lines. Time was significantly associated with decreasing rates of positive emotion (*b* = −0.003, *p* < .001), anxiety (*b* = −0.005, *p* < .001) and sadness (*b* = −0.001, *p* = .040). Relative changes were largest for anxiety, which decreased by 18.2% (positive emotion −9.4%, sadness −5.4%, anger 3.5%).

### Topic labels and associations with topics

Inspection of semantic coherence and exclusivity values suggested that a topic model with k = 30 topics provided an adequate fit to the data (see S2). Rates of emotion were significantly increased in a total of 16 topics (sees Tables 2, 3 and S3 for an overview of topics). These topics were labelled based on the 15 top words identified by the probability and FREX metric. For example, the topic characterized by the words ‘measures’ and ‘government’ (probability metric) and the words ‘fundamental rights’ and ‘citizen’ (FREX metric) was subsequently labelled ‘Political measures and implications for basic rights’ (see Table 2). The labelled topics could be categorized into four broader themes (see Table 3).

1. Measures to prevent the spread of Covid-19: Topics in this theme included tweets relating to the impact of political measures to prevent disease spread (e.g. lockdown) on basic rights, social contact restrictions and compliance with these measures, the opening and closing of schools and communication and actions of political leaders.
2. Life during lockdown: Topics in this theme included tweets associated with caring for loved ones, homeoffice and leisure at home, information and digital technologies as well as environment and nature.
3. Infection-related issues: Topics in this theme were generated by tweets covering symptoms, testing and quarantine related to Covid-19, infection risk as well as gratitude for healthcare workers.
4. Impact of pandemic on public and private life: One topic in this theme comprised tweets related to a relatively broad discussions about the impact of the pandemic on the economy and society in conjunction with other global crises (e.g. climate change). Other topics related to panic buying, conspiracy theories and (mis)information, religion as well as the pandemic situation in other world regions and populations, e.g. refugee camps.

**Table 2:** Words associated with topics

Topic label	Topic %^a^	Probability metric^b^	FREX metric^c^
Political measures and implications for basic rights	23.9	measures, government, germany, question, crisis, #merkel, #stayathome, economy, politics, opinion, country, population, politician, fear, citizen	fundamental rights, citizen, freedom, debate, restriction, relaxation, measures, restriction, citizen, democracy, containment, federal government, strategy, criticism, federal council
Social contact restrictions and compliance	9.1	#stayathome, distance, #curfew, city, #berlin, police, children, curfew, berlin, groups, work, home, germany, #contact ban, street	groups, parks, streets, police, city, park, street, bike, flat, pensioner, curfew, idiots, flats, windows, party
Closing and opening of schools	1.8	school, schools, #laschet, #school opening, pupils, #nrw, #final exams, #school, children, #schools, parents, teachers, #schoolboycottnrw, #school boycott ger, #loosening	#final exams, #school openings, pupils, #school opening, #school boycotts ger, #school, lessons, exams, #school boycott nrw, final exams, #gebauer, #average exams, high-school graduate, #schools, #school boycotts
Political leaders	1.9	#merkel, merkel, #cdu, #spd, #söder, #laschet, angela, #csu, krise, #fdp, #spahn, chancellor, söder, speeches, #afd	angela, #cdu, #fdp, #csu, federal chancellor, #spd, merkel, chancellor, söder, speech, #merkel, speech, cdu, merkels, prime ministers
Caring for loved ones	1.5	children, parents, child, family, children, home, #stayathome, mother, grandmother, daughter, school, father, friends, kita, mum	kita, grandpa, parents, playground, #kita, grandma, child, grandparents, children, grandchildren, mum, dad, daughter, emergency care
Homeoffice and leisure	18.2	#stayathome, #homeoffice, home quarantine, thank you, work, home office, children, world, evening, family, child, head, toilet paper, #quarantine	home office,office, #homeoffice, books, couch, film, netflix, living room, isolation, sofa, internet, flat, coffee, cat, bed
Information and digital technologies	4.5	questions, #homeoffice, crisis, #stayathome, article, app, #digitalization, thank you, contribution, work, tips, answer, infos, colleagues, company	#data protection, #digitisation, webinar, challenges, #newwork, digitisation, hackathon, apps, blog, tips, app, #hackathon, #app, google, teams
Environment and nature	0.9	#stayathome, air, world, sun, nature, weather, #ostern, rest, balcony, distance, #distancing, garden, #socialdistancing, crisis, dog	cats, nature, #notjustsad, sea, snow, cars, #rastatt, forest, #orchestra, dog, cycling, birds, #beethoven, #bathing, aeroplanes
Symptoms, testing and quarantine	2.2	test, quarantine, contact, symptoms, doctor, fever, patients, cough, tests, question, hospital, #stayathome, health authority, home, risk group	fever, cough, test, general practitioner, health office, symptoms, smear test, symptoms, result, contact, call, doctor, quarantine, risk region
Infection risk	1.8	anxiety, patients, hospital, death, risk group, home, relatives, mother, pre-existing conditions, worry, italy, thoughts, family, deaths, situation	respiration, preexisting conditions, relatives, course, age, condolence, smoker, lung, sick, death, intensive, fear, anxiety, risk groups, cancer
Gratitude for healthcare workers	1.9	thanks, doctors, #care, patients, colleagues, #stayathome, crisis, personnel, nurses, staff, coimmitment, masks, staff, care	nurse, #nursing, #nursing crisis, #nursing, #nursing, #nursing, heroes, #nursing, running, nurses, working conditions, payment, applause, care, #hospital, #system relevant
Economic and societal crisis	6.4	crisis, world, fear, economy, society, #stayathome, solidarity, germany, children, pandemic, country, politician, opinion, situation, future	#climate crisis, egoism, freedom, society, #fridaysforfuture, economy, climate, climate change, planet, chance, #climate change, #fff, future, humanity, thinking
Panic buying	1.8	loo paper, #loo paper, toilet paper, #toilet paper, flour, #hamster purchases, noodles, pack, #stayathome, supermarket, #hamster purchases, packs, rolls, yeast, bread	flour, pasta, loo paper, #hamster purchases, #hamster purchases, yeast, #toilet paper gate, packs, #flour, milk, toilet paper, pack, rolls, #loo paper, #toilet paper
Conspiracy theories and misinformation	1.3	#trump, trump, usa, #usa, boris, johnson, #johnson, #qanon, new, york, donald, world, #who, deepstatevirus, president	#deepstatevirus, #johnson, #deepstateexposed, #qanon, boris, trump, donald, #trump, #deepfake, johnson, #draintheswamp, #deepstatetakedown, #satanicelite, #trumpvirus, american
Religion	0.9	easter, #easter, #afd, church, #noafd, churches, #ramadan, jesus, #church, #stayathome, family, hope, love, power, afd	jesus, #ramadan, churches, christians, church, god, #prohibit afd now, prayer, #fckafd, nazis, mosques, #belief, #church, muslim, #noafd
Other affected regions and populations	2	germany, italy, china, europe, #italia, country, #eu, countries, world, usa, solidarity, #europe, #germany, #china, aid	europe, greece, #lesbos, russia, #eu, #leavenoonebehind, countries, iran, #moria, europe, #spain, #refugees, #greece, #europe, cuba

Top 15 words of topics that showed significant associations with an emotion; ^a^Proportion of tweets assigned to topic; ^b^Most probable words per topic; ^c^Words with highest FREX scores per topics, i.e. most probable words weighted by their exclusivity for a given topic

**Table 3:** Topics and associations with emotions

General theme	Topic	Topic %	Emotion (OR, 95% CI)			
			Anger	Anxiety	Positive emotion	Sadness
Measures to prevent spread of Covid-19	Political measures and implications for basic rights	23.9	1.12 (1.08–1.16)	1.28 (1.22–1.34)		
	Social contact restrictions and compliance	9.1	1.26 (1.2–1.33)			
	Closing and opening of schools	1.8		1.25 (1.08–1.42)		
	Political leaders	1.9			1.15 (1.1–1.19)	
Life during lockdown	Caring for loved ones	1.5		1.27 (1.09–1.47)		1.42 (1.28–1.58)
	Homeoffice and leisure	18.2			1.09 (1.07–1.1)	
	Information and digital technologies	4.5			1.03 (1–1.07)	
	Environment and nature	0.9			1.18 (1.11–1.25)	1.21 (1.04–1.4)
Infection	Symptoms, testing and quarantine	2.2		1.37 (1.22–1.54)		1.17 (1.06–1.29)
	Infection risk	1.8		2.77 (2.54–3.03)		1.82 (1.67–1.98)
	Gratitude for healthcare workers	1.9			1.22 (1.17–1.27)	
Impact of pandemic on public and private life	Economic, societal and other crisis	6.4	1.61 (1.53–1.7)	1.57 (1.47–1.68)	1.09 (1.06–1.12)	1.28 (1.21–1.35)
	Panic buying	1.8	1.31 (1.18–1.45)			
	Conspiracy theories and misinformation	1.3	1.43 (1.28–1.6)			1.2 (1.05–1.35)
	Religion	0.9			1.17 (1.1–1.24)	1.32 (1.14–1.52)
	Other affected regions and populations	2				1.21 (1.09–1.34)

Topics relating to the theme ‘Measures to prevent spread of Covid-19’ were mostly related to anger and anxiety, although discussions of political leaders were associated with positive emotions. The majority of topics belonging to the theme ‘life during lockdown’ were associated with positive emotions. Only tweets belonging to the topic of caring for loved ones were associated with anxiety and sadness. Topics included in the theme ‘infection’ were mostly associated with anxiety and sadness, except for the topic gratitude for healthcare workers, which was associated with positive emotions. Most topics covered by the theme ‘impact of pandemic on public and private life’ were associated with anxiety, anger or sadness.

Topics most strongly associated with either anger, anxiety, sadness or positive emotion comprised 18.6%, 29.7%, 5.3% and 26.5% of all tweets, respectively. The remaining 19.9% of tweets were assigned to topics not significantly related to any emotion.

## Discussion

Non-pharmaceutical interventions such as social contact restrictions and lockdowns have proven effective in slowing the spread of Covid-19; however, little is known about the psychological responses of the public to the pandemic and efforts to control it (Hsiang et al. 2020). We found that rates of anxiety, sadness and positive emotion in Twitter statements decreased over a 1-month period following the introduction of social contact restrictions in German-speaking countries of Central Europe, suggesting reduced polarity of emotional expressions in tweets. Several topics emerged in Twitter discourse that were related to implications of the Covid-19 pandemic in general and to the introduction of social contact restrictions specifically.

Cautious interpretation of these results is warranted, as an individual’s use of words may not precisely reflect underlying psychological states. While several findings show that measures of subjective well-being or mental health are associated with differences in language use, other studies did not find a relationship between psychological measures and language use (Meier et al. 2019; Sonnenschein et al. 2018). Thus, results of linguistic analyses such as this study should be interpreted in conjunction with other indicators of public well-being. In this study, rates of words related to anxiety showed the largest decrease over time. This result may suggest a possible link between governmental efforts to control the pandemic and decreasing anxiety among the public (Luo et al. 2020). In Germany, for example, public approval of social contact restrictions was high at the time of their introduction and Twitter statements concerning political leaders were associated with more positive emotion in this study (Teufel et al. 2020). However, no causal link between governmental interventions and rates of emotions in Twitter statements can be established due to the observational design of this study and the lack of a control group.

It is also possible that decreasing rates of anxiety-related words in Twitter statements reflect a habituation or emotional numbness to pandemic stressors. Topics related to anxiety were most prevalent in this sample. These results corroborate other research showing heightened levels of anxiety in populations affected by Covid-19 (Wang et al. 2020). Congruent with findings from other studies, sources of anxiety and sadness were mainly related to the impact of the pandemic on family and social life, education, as well as infection, pointing to an need to address these public concerns (Mertens et al. 2020; Abd-Alrazaq et al. 2020). With regard to social contact restrictions, anxiety was associated with topics discussing potential negative impacts of both implementation (e.g. with regard to basic rights) and relaxation of social contact restrictions (e.g. re-opening of schools), which suggests nuanced public risk perceptions.

Personal statements expressing positive emotions declined steadily following the introduction of social contact restrictions. Psychological strain resulting from enduring social isolation in addition to the absence of directly observable effects of prevention efforts (e.g. avoiding increasing rather than reducing case-fatality rates) may have inhibited positive emotions. However, topics relating to positive emotions comprised approximately one-quarter of all tweets, indicating that social networks constitute an important source for people to share positive messages of coping with staying at home or solidarity (e.g. with healthcare workers).

Expressions of anger did not change in tweets posted after the introduction of social contact restrictions. Anger was related to social contact restrictions and compliance with these measures, but it remains unclear whether Twitter users expressed anger about having to comply with restrictions or about other people not complying. Anger was also related to statements surrounding the credibility of Covid-19-related information. Misinformation on social media has been identified as a major barrier for public health efforts to prevent disease spread, promoting unhealthy behaviour and erroneous practices that can ultimately harm physical and mental health (Tasnim et al. 2020). The association with anger observed in this study may indicate increasing public awareness and rejection of misinformation, but also highlights the need for concerted efforts strengthening the position of authentic sources of information.

### Limitations

Several limitations to this study need to be considered. The target population of this study were Twitter users living in a country in German-speaking Central Europe, but we were not able to determine the exact origin of the majority of tweets. However, user-tagged country of origin indicated that the vast majority of tweets originated from users in Germany, Switzerland or Austria. The following issues specifically limit the generalizability of results: First, we used one of Twitter’s free data access point for which Twitter does not provide all tweets matching a certain search query but samples a collection of tweets with undisclosed underlying sampling scheme. Second, we used a hashtag-based search to identify relevant tweets, seeking to increase the relevance of tweets obtained via Twitter’s data access point. However, samples of tweets collected by hashtag-based searches may not be representative of discourse related to a specific topic (Bruns and Stieglitz 2014). Third, compared to the general population, Twitter users have been shown to be younger, higher educated and more politically liberal (Mellon and Prosser 2017). Last, there was indication that the vast majority of the tweets were posted by German users, followed by users from Switzerland and Austria (97.2% for the three countries combined), which limits the generalizability of results to other countries. Although the timing and stringency of social contact restrictions within Germany, Switzerland and Austria were largely comparable (Hale et al. 2020), different experiences within these populations may have also reduced generalizability of results across these countries.

Furthermore, the word count approach we used to estimate the prevalence of emotions in our sample did not allow identifying negations and other contextual shifters of word meanings (e.g. sarcasm). However, we calculated rates of emotions based on aggregated tweets only (i.e. at the overall or daily level), and thus single misclassifications are unlikely to bias overall results. Observed changes in rates of emotion in Twitter discourse may represent lagged effects of events other than the introduction of social contact restrictions. We were not able to collect data for 1 day in the study period. However, bias resulting from this minimal lack of data is likely to be negligible.

### Conclusion

Polarity of emotions expressed by Twitter users decreased following the introduction of social contact restrictions in countries of German-speaking central Europe, mainly driven by decreases in anxiety. Findings further revealed nuanced emotional responses to different aspects of social contact restrictions and the pandemic in general. Providing the public with accessible rationales for both implementation and relaxation of social contact restrictions as well as reliable information on epidemiological and medical aspects of the virus may buffer negative emotions. Continuous monitoring of social media activity at a regional level can contribute to adaptive understanding and responses to changing public concerns over the course of the pandemic.

## Figures and Tables

**Fig. 2:**
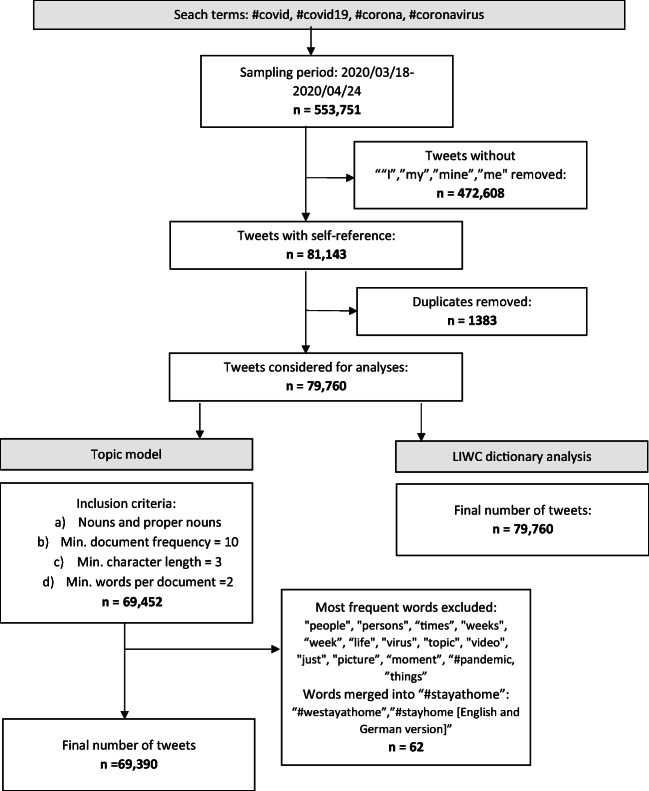
Sample flowchart

**Fig. 3:**
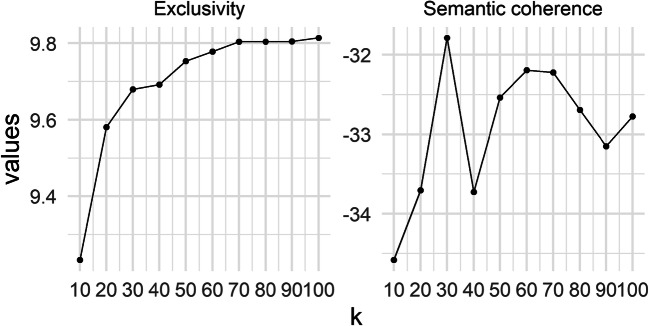
Average exclusivity and semantic coherence models with k-topics

## Data Availability

Unique identifiers of tweets which may be used to retrieve the full content of tweets included in the analyses are available from the authors upon request.

## Appendix

### S2. Model selection process

Average exclusivity values showed the largest increases for the model with 30 topics, after which trends appeared to flatten out (see Fig. 3). Average semantic coherence was highest for the model with 30 topics. Thus, we chose the model with 40 topics for further analyses.

### S3. Topic labels, proportions and associated words

**Table:**

Topic Nr	Topic label^a^	%^b^	Topic words: German (original)		Topic words: English	
			Probability metric^c^	FREX metric^d^	Probability metric^c^	FREX metric^d^
1	Caring for loved ones	1.5	kinder, eltern, kind, familie, kindern, hause, #stayathome, mutter, oma, tochter, schule, vater, freunde, kita, mama	kita, opa, eltern, spielplatz, #kita, oma, kind, kindern, großeltern, kinder, enkel, mama, papa, tochter, notbetreuung	children, parents, child, family, children, home, #stayathome, mother, grandmother, daughter, school, father, friends, kita, mum	kita, grandpa, parents, playground, #kita, grandma, child, grandparents, children, grandchildren, mum, dad, daughter, emergency care
2	Closing and opening of schools	1.8	schule, schulen, #laschet, #schuloeffnung, schüler, #nrw, #abitur, #schule, kinder, #schulen, eltern, lehrer, #schulboykottnrw, #schulboykottde, #lockerungen	#abitur, #schuloeffnung, schüler, #schuloeffnungen, #schulboykottde, #schule, unterricht, prüfungen, #schulboykottnrw, abitur, #gebauer, #durchschnittsabitur, abiturienten, #schulen, #schulboykott	school, schools, #laschet, #school opening, pupils, #nrw, #final exams, #school, children, #schools, parents, teachers, #schoolboycottnrw, #school boycott ger, #loosening	#final exams, #school openings, pupils, #school opening, #school boycotts ger, #school, lessons, exams, #school boycott nrw, final exams, #gebauer, #average exams, high-school graduate, #schools, #school boycotts
3	Social contact restrictions and compliance	9.1	#stayathome, abstand, #ausgangssperre, stadt, #berlin, polizei, kinder, ausgangssperre, berlin, gruppen, arbeit, hause, deutschland, #kontaktverbot, straße	gruppen, parks, straßen, polizei, stadt, park, straße, rad, wohnung, rentner, ausgangssperre, idioten, wohnungen, fenster, party	#stayathome, distance, #curfew, city, #berlin, police, children, curfew, berlin, groups, work, home, germany, #contact ban, street	groups, parks, streets, police, city, park, street, bike, appartment, pensioner, curfew, idiots, flats, windows, party
4	Conspiracy theories and misinformation	1.3	#trump, trump, usa, #usa, boris, johnson, #johnson, #qanon, new, york, donald, welt, #who, #deepstatevirus, präsident	#deepstatevirus, #johnson, #deepstateexposed, #qanon, boris, trump, donald, #trump, #deepfake, johnson, #draintheswamp, #deepstatetakedown, #satanicelite, #trumpvirus, amerikaner	#trump, trump, usa, #usa, boris, johnson, #johnson, #qanon, new, york, donald, world, #who, #deepstatevirus, president	#deepstatevirus, #johnson, #deepstateexposed, #qanon, boris, trump, donald, #trump, #deepfake, johnson, #draintheswamp, #deepstatetakedown, #satanicelite, #trumpvirus, american
5	Environment and nature	0.9	#stayathome, luft, welt, sonne, natur, wetter, #ostern, ruhe, balkon, abstand, #abstandhalten, garten, #socialdistancing, krise, hund	katzen, natur, #notjustsad, meer, schnee, autos, #rastatt, wald, #orchester, hund, radfahren, vögel, #beethoven, #baden, flugzeuge	#stayathome, air, world, sun, nature, weather, #ostern, rest, balcony, distance, #distancing, garden, #socialdistancing, crisis, dog	cats, nature, #notjustsad, sea, snow, cars, #rastatt, forest, #orchestra, dog, cycling, birds, #beethoven, #bathing, aeroplanes
6	Economic, societal and other crisis	6.4	krise, welt, angst, wirtschaft, gesellschaft, #stayathome, solidarität, deutschland, kinder, pandemie, land, politiker, meinung, situation, zukunft	#klimakrise, egoismus, freiheit, gesellschaft, #fridaysforfuture, wirtschaft, klima, klimawandel, planeten, chance, #klimawandel, #fff, zukunft, menschheit, denk	crisis, world, fear, economy, society, #stayathome, solidarity, germany, children, pandemic, country, politician, opinion, situation, future	#climate crisis, egoism, freedom, society, #fridaysforfuture, economy, climate, climate change, planet, chance, #climate change, #fff, future, humanity, thinking
7	Gratitude for healthcare workers	1.9	danke, arbeit, ärzte, #pflege, patienten, kollegen, #stayathome, krise, personal, pfleger, pflegekräfte, einsatz, masken, mitarbeiter, pflege	pfleger, #pflegenotstand, pflegekräfte, #pflege, #pflegekräfte, helden, #pflegekraefte, laufen, krankenschwestern, arbeitsbedingungen, bezahlung, applaus, pflege, #krankenhaus, #systemrelevant	thanks, doctors, #care, patients, colleagues, #stayathome, crisis, personnel, nurses, staff, coimmitment, masks, staff, care	nurse, #nursing, #nursing crisis, #nursing, #nursing, #nursing, heroes, #nursing, running, nurses, working conditions, payment, applause, care, #hospital, #system relevant
8	Other affected regions and populations	2	deutschland, italien, china, europa, #italien, land, #eu, länder, welt, usa, solidarität, #europa, #deutschland, #china, hilfe	europa, griechenland, #lesbos, russland, #eu, #leavenoonebehind, länder, iran, #moria, europas, #spanien, flüchtlinge, #griechenland, #europa, kuba	germany, italy, china, europe, #italia, country, #eu, countries, world, usa, solidarity, #europe, #germany, #china, aid	europe, greece, #lesbos, russia, #eu, #leavenoonebehind, countries, iran, #moria, europe, #spain, #refugees, #greece, #europe, cuba
9	Homeoffice and leisure	18.2	#stayathome, #homeoffice, hause, quarantäne, danke, arbeit, homeoffice, kinder, welt, abend, familie, kind, kopf, klopapier, #quarantaene	homeoffice, büro, #homeoffice, bücher, couch, film, netflix, wohnzimmer, isolation, sofa, internet, wohnung, kaffee, katze, bett	#stayathome, #homeoffice, home quarantine, thank you, work, home office, children, world, evening, family, child, head, toilet paper, #quarantine	home office,office, #homeoffice, books, couch, film, netflix, living room, isolation, sofa, internet, flat, coffee, cat, bed
10	Infection risk	1.8	angst, patienten, krankenhaus, tod, risikogruppe, hause, angehörigen, mutter, vorerkrankungen, sorgen, italien, gedanken, familie, tote, situation	beatmung, vorerkrankungen, angehörigen, verlauf, alter, beileid, raucher, lunge, kranke, tod, intensiv, angst, ängste, risikogruppen, krebs	anxiety, patients, hospital, death, risk group, home, relatives, mother, pre-existing conditions, worry, italy, thoughts, family, deaths, situation	respiration, preexisting conditions, relatives, course, age, condolence, smoker, lung, sick, death, intensive, fear, anxiety, risk groups, cancer
11	Information and digital technologies	4.5	fragen, #homeoffice, krise, #stayathome, artikel, app, #digitalisierung, danke, beitrag, arbeit, tipps, antworten, infos, kollegen, unternehmen	#datenschutz, #digitalisierung, webinar, herausforderungen, #newwork, digitalisierung, hackathon, apps, blog, tipps, app, #hackathon, #app, google, teams	questions, #homeoffice, crisis, #stayathome, article, app, #digitalization, thank you, contribution, work, tips, answer, infos, colleagues, company	#data protection, #digitisation, webinar, challenges, #newwork, digitisation, hackathon, apps, blog, tips, app, #hackathon, #app, google, teams
12	Panic buying	1.8	klopapier, #klopapier, toilettenpapier, #toilettenpapier, mehl, #hamsterkaeufe, nudeln, packung, #stayathome, supermarkt, #hamsterkäufe, packungen, rollen, hefe, brot	mehl, nudeln, klopapier, #hamsterkaeufe, #hamsterkäufe, hefe, #klopapiergate, packungen, #mehl, milch, toilettenpapier, packung, rollen, #klopapier, #toilettenpapier	loo paper, #loo paper, toilet paper, #toilet paper, flour, #hamster purchases, noodles, pack, #stayathome, supermarket, #hamster purchases, packs, rolls, yeast, bread	flour, pasta, loo paper, #hamster purchases, #hamster purchases, yeast, #toilet paper gate, packs, #flour, milk, toilet paper, pack, rolls, #loo paper, #toilet paper
13	Political leaders	1.9	#merkel, merkel, #cdu, #spd, #söder, #laschet, angela, #csu, krise, #fdp, #spahn, kanzlerin, söder, rede, #afd	angela, #cdu, #fdp, #csu, bundeskanzlerin, #spd, merkel, kanzlerin, söder, rede, #merkel, ansprache, cdu, merkels, ministerpräsidenten	#merkel, merkel, #cdu, #spd, #söder, #laschet, angela, #csu, krise, #fdp, #spahn, chancellor, söder, speeches, #afd	angela, #cdu, #fdp, #csu, federal chancellor, #spd, merkel, chancellor, söder, speech, #merkel, speech, cdu, merkels, prime ministers
14	Political measures and implications for basic rights	23.9	maßnahmen, regierung, deutschland, frage, krise, #merkel, #stayathome, wirtschaft, politik, meinung, land, bevölkerung, politiker, angst, bürger	grundrechte, freiheit, debatte, einschränkungen, lockerung, maßnahmen, einschränkung, massnahmen, bürger, demokratie, eindämmung, bundesregierung, strategie, kritik, bundesrat	measures, government, germany, question, crisis, #merkel, #stayathome, economy, politics, opinion, country, population, politician, fear, citizen	fundamental rights, citizen, freedom, debate, restriction, relaxation, measures, restriction, citizen, democracy, containment, federal government, strategy, criticism, federal council
15	Religion	0.9	ostern, #ostern, #afd, kirche, #noafd, kirchen, #ramadan, jesus, #kirche, #stayathome, familie, hoffnung, liebe, kraft, afd	jesus, #ramadan, kirchen, christen, kirche, gottes, #afdverbotjetzt, gebet, #fckafd, nazis, moscheen, #glaube, #kirche, muslime, #noafd	easter, #easter, #afd, church, #noafd, churches, #ramadan, jesus, #church, #stayathome, family, hope, love, power, afd	jesus, #ramadan, churches, christians, church, god, #prohibit afd now, prayer, #fckafd, nazis, mosques, #belief, #church, muslim, #noafd
16	Symptoms, testing and quarantine	2.2	test, quarantäne, kontakt, symptome, arzt, fieber, patienten, husten, tests, frage, krankenhaus, #stayathome, gesundheitsamt, hause, risikogruppe	fieber, husten, test, hausarzt, gesundheitsamt, symptome, abstrich, symptomen, ergebnis, kontakt, anruf, arzt, ärztin, quarantäne, risikogebiet	test, quarantine, contact, symptoms, doctor, fever, patients, cough, tests, question, hospital, #stayathome, health authority, home, risk group	fever, cough, test, general practitioner, health office, symptoms, smear test, symptoms, result, contact, call, doctor, quarantine, risk region
17		3	geld, krise, unternehmen, euro, hilfe, staat, wirtschaft, kurzarbeit, frage, mitarbeiter, firma, kosten, firmen, danke, unterstützung	firmen, #soforthilfe, soforthilfe, kredite, antrag, #kurzarbeit, freiberufler, hilfen, steuern, künstler, staat, kurzarbeit, #bge, banken, gutscheine	money, crisis, company, clients, euro, economy, short time, employee, company, short time work, help, state, question, employee, thank you, support	companies, #emergency aid, emergency aid, loans, application, #short work, freelancer, help, taxes, state, short work, #bge, banks, vouchers, artist
18		2.8	zahlen, deutschland, zahl, tote, italien, fälle, daten, anzahl, tests, infizierten, toten, maßnahmen, todesfälle, #deutschland, usa	zahl, einwohner, todesfälle, zahlen, anzahl, anstieg, neuinfektionen, fallzahlen, infizierten, tote, hopkins, vergleich, wachstum, infektionen, fälle	numbers, germany, number, dead, italy, cases, data, number, tests, infected, dead, measures, deaths, #germany, usa	number, population, deaths, numbers, number, increase, increase, number, new infections, number of cases, infected, fatalities, hopkins, comparison, growth, infections, cases
19		2.2	danke, medien, informationen, fragen, twitter, #stayathome, youtube, artikel, nachrichten, facebook, situation, frage, #fakenews, videos, beitrag	facebook, zdf, berichterstattung, informationen, sender, verschwörungstheorien, youtube, fakten, vogt, account, journalismus, twitter, radio, sendung, qualität	thanks, media, information, questions, twitter, #stayathome, youtube, article, news, facebook, situation, question, #fakenews, videos, contribution	facebook, zdf, broadcast, information, broadcaster, conspiracy theories, youtube, facts, vogt, account, journalism, twitter, radio, reporting, quality
20		2	dr., prof., #rki, grippe, impfstoff, #drosten, experten, drosten, #lanz, meinung, virologen, virologe, viren, studie, interview	prof., drosten, #drosten, virologe, streeck, dr., impfstoff, #wieler, wieler, bhakdi, impfung, experten, #streeck, virologen, experte	dr., prof., #rki, influenza, vaccine, #drosten, expert, drosten #lanz, opinion, virologist, virologist, virus, study, interview	prof., drosten, #drosten, virologist, streeck, dr., vaccine, #wieler, wieler, bhakdi, vaccination, experts, #streeck, virologist, expert
21		2	#stayathome, #flattenthecurve, hause, #ausgangssperre, #homeoffice, #socialdistancing, #bleibtgesund, #zuhausebleiben, zuhause, #stayhomesavelives, #bleibzuhause, #staysafe, danke, #ostern, #quarantaene	#stayathomechallenge, bleibt, #stayhomesavelives, #wirbleibendaheim, #staythefuckhome, #wirschaffendas, #bleibtgesund, #zu, #flattenthecurve, #flattenthecuve, hausebleiben, universum, #wirbleibenzu, #staysafe, #deutschlandmachtmusik	#stayathome, #flattenthecurve, home #curfew, #homeoffice, #socialdistancing, #stayhealthy, #stay at home, #stayhomesavelives, #stay at home, #staysafe, thank you, #ostern, #quarantine	#stayathomechallenge, stay, #stayhomesavelives, #westayathome, #staythefuckhome, #wewillmakeit, #stayhealthy, #zu, #flattenthecurve, #flattenthecuve, homestay, universe, #westayat, #staysafe, #germanymakesmusic
22		2	abstand, supermarkt, kasse, kunden, hände, mundschutz, einkaufswagen, hand, meter, maske, handschuhe, laden, gesicht, einkaufen, paket	einkaufswagen, kasse, meter, schlange, wagen, unterschrift, eingang, kunde, mindestabstand, handschuhe, ware, stift, aldi, kunden, verkäuferin	distance, supermarket, cash desk, customers, hands, mouth protection, shopping cart, hand, meter, mask, gloves, load, face, shopping, package	shopping cart, cash desk, meter, queue, trolley, signature, entrance, customer, minimum distance, gloves, merchandise, pen, aldi, customer, salesperson
23		1.8	masken, maske, #maskenpflicht, mundschutz, #mundschutz, #maskeauf, #masken, #maske, nase, pflicht, mund, tragen, maskenpflicht, schutz, #schutzmasken	#maskeauf, tragen, #mundschutzpflicht, pflicht, nähmaschine, #mundschutzmasken, brille, #maske, #mundschutz, masken, maske, #plauen, #vogtland, #faceshield, #masken	masks, mask, #mask obligation mouth-protection, #mouth-protection, #mask on, #mask, #masks,nose, obligation, mouth, wearing, mask oblgation protection, #protective masks	#mask on, wearing, #mask obligation, obligation, sewing machine, #mask, glasses, #mask, #mask, #mask, masks, mask, #plauen, #vogtland, #faceshield, #masks
24		1.1	#deutschland, #stayathome, #politik, #wirtschaft, #gesundheit, #krise, #virus, #merkel, #zdf, #gesellschaft, #exitstrategie, #medien, #maskenpflicht, #lanz, #solidarität	#wirtschaft, #gesellschaft, #gesundheit, #freiheit, #bürger, #politik, #exitstrategie, #medien, #wissenschaft, #exit, #börse, #panik, #menschen, #zdf, #krise	#germany, #stayathome, #politics, #economy, #health, #crisis, #virus, #merkel, #zdf, #society, #exit strategy, #media, #mask obligation, #lanz, #solidarity	#economy, #society, #health, #freedom, #citizens, #politics, #exit strategy, #media, #science, #exit, #stock exchange, #panic, #people, #zdf, #crisis
25		1.1	april, mai, anfang, schulen, mitte, #stayathome, geschäfte, bayern, märz, urlaub, lockerungen, montag, #lockdown, august, maßnahmen	august, mitte, april, großveranstaltungen, restaurants, friseure, veranstaltungen, mai, juni, friseur, konzerte, oktoberfest, september, hotels, bars	april, may, start, schools, middle, #stayathome, shops, bavaria, march, holiday, loosening, monday, #lockdown, august, measures	august, middle, april, large public events, restaurants, hairdressers, events, may, june, hairdresser, concerts, oktoberfest, september, hotels, bars
26		0.7	#stayathome, #youtube, #twitch, #twitchde, #gaming, #streamer, spaß, #livestream, #live, #letsplay, #stream, #streaming, #video, #twitchstreamer, kanal	#streamer, #twitchde, #twitch, #gaming, #letsplay, #twitchstreamer, #stream, #twitchtv, #livestream, #rap, #streaming, #youtuber, #live, #supportsmallstreamers, #germanmediart	#stayathome, #youtube, #twitch, #twitchde, #gaming, #streamer, fun, #livestream, #live, #letsplay, #stream, #streaming, #video, #twitchstreamer, chanel	#streamer, #twitchde, #twitch, #gaming, #letsplay, #twitchstreamer, #stream, #twitchtv, #livestream, #rap, #streaming, #youtuber, #live, #supportsmallstreamers, #germanmediart
27		0.6	#bundesliga, #kurz, fußball, #dfl, #ischgl, #övp, #tirol, #fussball, #geisterspiele, #österreich, #gruenen, österreich, #kogler, #tuerkisgruen, spieler	#fussball, #dfl, #tuerkisgruen, #bundesliga, #kogler, #geisterspiele, #dfb, fußball, #oevp, #anschober, #platter, #tirol, geisterspiele, saison, fussball	#bundesliga, #kurz, football, #dfl, #ischgl, #övp, #tirol, #soccer, #ghost games, #austria, #gruenen, austria, #kogler, #turquoise green, player	#soccer, #dfl, #turquoise green, #premiereleague, #kogler, #ghost games, #dfb, football, #oevp, #anschober, #platter, #tirol, ghost games, season, soccer
28		0.3	#adidas, miete, #deichmann, himmel, #online, handy, beitrag, #miete, adidas, #letzebuerg, #hundm, #info, #news, #media, vermieter	#letzebuerg, #deichmann, #adidas, #hundm, adidas, spiegelreflexkamera, witterung, #info, #letzebuergesch, #media, #miete, #moien, #online, miete, h&	#adidas, rent, #deichmann, sky, #online, mobile phone, contribution, #rent, adidas, #letzebuerg, #hundm, #info, #news, #media, landlord	#letzebuerg, #deichmann, #adidas, #hundm, adidas, reflex camera, weather, #info, #letzebuergesch, #media, #rent, #moien, #online, rent, h&
29		0.2	#bayern, #sz, #faz, #zeit, #nzz, #spiegelonline, #oktoberfest, #wiesn, #zahlen, wiesn, #söder, #china, #markussoeder, #münchen, #umwelt	#sz, #nzz, #zeit, #spiegelonline, #faz, #zahlen, wiesn, #wiesn, #bayern, #markussoeder, beate, #oktoberfest, harald, #muenchen, #bahner	#bayern, #sz, #faz, #zeit, #nzz, #spiegelonline, #oktoberfest, #wiesn, #numbers, wiesn, #söder, #china, #markussoeder, #münchen, #environment	#sz, #nzz, #zeit, #spiegelonline, #faz, #numbers, wiesn, #wiesn, #bavaria, #markussoeder, beate, #oktoberfest, harald, #muenchen, #bahner
30		0.2	#quarantäne, #türkisgrün, #dlvö, #hausarrest, #wien, #teamgoaschtig, wien, wasser, till, kamera, #bilderrätsel, hause, #meidling, #kurz, #wienliebe	#dlvö, #teamgoaschtig, #türkisgrün, #hausarrest, #bilderrätsel, #meidling, till, #wienliebe, kamera, chuck, norris, #rammstein, #quarantäne, joggis, #maisach	#quarantine, #turquoise green, #dlvö, #house arrest, #wien, #teamgoaschtig, vienna, water, till, camera, #picture puzzle, home, #meidling, #kurz, #love vienna	#dlvö, #teamgoaschtig, #turquoise-green, #house arrest, #picture puzzles, #meidling, till, #love wien, camera, chuck, norris, #rammstein, #quarantine, joggis, #maisach

20 top words per topic shown; ^a^Topic labels were only assigned to topics with significant association with an emotion; ^b^Proportion of all tweets; ^c^Most probable words per topic; ^d^Words with highest FREX scores per topics, i.e. most probable words weighted by their exclusivity for a given topic

## Code availability

The code underlying the statistical analyses is available from the first author on request.
